# The Diagnosis of Pyelonephritis

**Published:** 1907-08

**Authors:** 


### THE DIAGNOSIS OF PYELONEPHRITIS.

In a preliminary communication E. Beer publishes the histories of two cases which seem to point to a new diagnostic sign of the involvment of the kidney parenchyma. In the first case the patient discharged methylene blue stained pus in the urine at intervals up to two and three-fourths years after the last administration of the drug. The second patient had pyelonephritis (verified by nephrotomy) and had recurrent discharges of methylene stained pus over a month after the last aministration of the drug. He is of the opinion that the pigment is fixed in the pus in great part, if not wholly, in the leuco form and that its presence in the abcesses is the result of the excretion of the renal epithelial cells. He produced a pyelonephritis in a dog and then for several days administered methylene blue. The kidney was removed two weeks later and, after treating with oxidizing agents, showed multiple bluish gray pus foci throughout its substance. He summarizes his conclusions derived from the facts so far as follows: 1. There is no differential diagnostic sign between simple pylitis and pyelonephritis. 2. Pyuria from the upper urinary tract may be due to either of these conditions. 3. By the use of the above described methylene blue test it would seem that a different diagnosis may be made. 4. Methylene blue is deposited in the parenchymatous abscesses and may be stored in these for years. 5. A later discharge of methylene blue, bound to the pus, is indicative of the rupture of such parenchymatous abscesses into the pelvis of the kidney and is consequently diagnostic of pyelomphritis—Jour. A. M. A.

